# The Book World of Medicine and Science

**Published:** 1899-10-21

**Authors:** 


### The Book World of Medicine and Science.

Medical Gymnastics, including the Schott (Nauheim) Movements; being a Text-Book of Massage and Mechanical Therapeutics Generally. By Axel V. Grafstrom, B.Sc., M.D. With eleven pen-and-ink sketches by the author. (London: Scientific Press. 1899. Price 2s. 6d.)

Movement is the very essence of man's healthy being, and, on the other hand, interference with motility is apt to be one of the first symptoms of disease. It is not, therefore, to be wondered at that some should have thought that it would be well for treatment of disease to be directed to the development of man's motor powers, to the restoration of a function which is known to be essential to the maintenance of health; and, indeed, practical experience seems to show that by mechano-therapy, as the "movement cure" is called, much may be done besides mere loosening of stiff joints and development of disused and paralysed muscles.

Dealing with the matter in a somewhat popular style, the author of this little book first gives an outline of the general effects of movement, whether active or passive; then he describes the various sorts of movements which are employed in mechano-therapy; after this he deals with massage, describing the process and its various modifications; and finally, in a series of chapters, he details the effects of these forms of treatment in the various classes of disease in which they are most commonly employed, and lays down rules as to the procedures most likely to be found beneficial in the several diseases mentioned. On the whole the recommendations given seem reasonable. The whole book is cut up into numbered paragraphs, with headings and sub-headings, in a manner which makes it very disagreeable to read, but this, we suppose, is necessary, as we notice that almost all massage books are affected with the same vice—or virtue, whichever it may be. As a clear exposition the book is certainly a good one, and will doubtless be found a useful handbook to mechano-therapy. If we were to raise any serious objection to books of this kind it would be that in trying to explain abstruse matters in simple language their writers are apt to get out of their depth, and to make somewhat positive statements which can hardly be supported by evidence.

The following recipe for getting a small baby instead of a large one, and thus ensuring an easy labour, seems likely to serve as an interesting and attractive bait to many readers of the gentler sex. It is reasonable enough to believe that the healthier the mother the more normal will be the child; but as to the explanation given the less said the better:—"Occasionally we meet women with an excellent physique, who, during pregnancy, and without suffering from any disease, lose flesh and strength. Such a woman grows emaciated and anæmic, and after a difficult and abnormal labour gives birth to a very large child. Her pelvis may be entirely normal. In such cases, and having the experience of a previous labour to govern us, a course of mechano-therapy will often improve the situation. The object of the treatment should be to increase the action of the organs of nutrition, and to carry the increased nutrition towards the mother's muscular system; that is, from within outward. By this the development of the fœtus will be retarded, and after a normal and comparatively easy labour a normal-sized child will be born."

What to our mind is a defect in this book, although we have no doubt that to some it will appear to be an extremely admirable arrangement, is the amount of space which is devoted to description, diagnosis, and treatment of disease, apart altogether from the details of mechano-therapy. Thus we find an article of about seven pages in length upon the subject of displaced kidney—rather an interesting article indeed—about six pages of which are devoted to etiology, diagnosis, and general treatment, and somewhat less than one page to the mechano-therapeutic methods to be used for its relief. Doubtless the book is made more interesting by this arrangement, but we should have thought that medical gymnastics ought by themselves to have been sufficiently important a theme to have enabled the author to have filled up his 134 pages without wandering into general medicine.

In a future edition perhaps it would be as well to eliminate the paragraph describing the treatment of strangulated hernia by massage. Strangulated hernia is a condition which should come under the care of a surgeon without delay, and this book is clearly addressed to those who are not surgeons. The operator, indeed, is told to "be on the look out for reduction *en masse* and rupture of the intestine, which are accidents greatly to be feared." Perhaps he would do better to look out for a surgeon. A paragraph describing the treatment of chronic seminal vesiculitis by massage of the seminal vesicles, *viâ* the rectum, seems to us to be almost as much out of place as the statement in another part of the book that the habit of habitual abortion is often remedied by pelvic massage. If, as is admitted in the preface, the book is not intended for "experts," these things are quite inadmissible.

